# Influence of Malting on Volatile Composition and Technological Properties of Polish Pea Varieties

**DOI:** 10.3390/foods14183224

**Published:** 2025-09-17

**Authors:** Alan Gasiński, Witold Pietrzak, Joanna Śniegowska, Joanna Kawa-Rygielska

**Affiliations:** Department of Fermentation and Cereals Technology, Wrocław University of Environmental and Life Sciences, Chełmońskiego 37, 51-630 Wrocław, Poland; witold.pietrzak@upwr.edu.pl (W.P.); joanna.sniegowska@upwr.edu.pl (J.Ś.); joanna.kawa-rygielska@upwr.edu.pl (J.K.-R.)

**Keywords:** germination, steeping, drying, mashing, volatiles, saccharification

## Abstract

Twelve Polish pea varieties were malted, and their technological properties were assessed, using a congress mashing regime. Alpha-amylase addition was also used to determine whether external enzymes can improve the properties of the achieved worts. Malts from peas were characterized with poor saccharification time, but alpha-amylase allowed for the saccharification of starch from three different pea varieties (‘Gloriosa’, ‘Jantar’, and ‘Primavil’). Acquired worts were characterized with a low amount of fermentable sugars, which would be inadequate for production of beer with the typical alcohol content (4–6% *v*/*v*), albeit they possibly could be used as a substrate for the production of low-alcoholic beers. Additionally, the malting process changed the amount and the type of volatiles present in the pea malts, significantly increasing the concentration of pyrazines, while, at the same time, reducing concentration of terpenes.

## 1. Introduction

Malting is a technological process typically used to modify grains of cereals, primarily barley. Malting constitutes seed hydration (steeping), germination, and drying (kilning). The main use of malt is the production of wort, which is then fermented to acquire beer. The main goal of malting is to increase the enzymatic activity of the malted seeds, but malts are also more friable, have a darker color, and are characterized by a modified aroma and taste [1,2]. In the recent years, the research about malted seeds reached different seeds than cereals, such as pseudocereals and even legumes [3,4,5,6]. Using malting to modify legume seeds seems very interesting, as legume seeds are one of the most important sources of protein and calories worldwide [7]. Malting can reduce the variety of components present in the legume seeds, which are characterized as anti-nutritional factors, such as phytic acid or raffinose-family oligosaccharides [8,9]. Malting can also significantly change the volatile composition of the substrate, as was presented in the case of lentils (*Lens culinaris*) [10]. Malted chickpea seeds (*Cicer arietinum*) can be used to produce hummus with a modified odor [11]. However, the previous research about the technological properties of malted legumes has shown that malts from legume seeds are typically characterized by worse filtration time and saccharification time, and the amount of wort acquired is definitely lower than when mashing barley malt. Wort was also characterized by lower extract content, and starch in most of the mashes produced from the legume malts did not saccharify completely. Long filtration time, low extract content, and low wort volume typically disqualify malts from being used to produce beer, due to low profitability. Most of the differences are the result of differences between the amount and activity of enzymes present in the barley, as well as the fact that barley contains a rather low amount of protein and a large amount of starch. Additionally, the presence of the barley husk significantly improves wort filtration. However, malts produced from peas (*Pisum sativum*) were one of the samples, which were characterized as one of the best properties (out of legume malts), as were lentils [5]. Subsequent analyses of lentil malts have shown that the malting procedure could be modified by increasing seed moisture content and germination temperature, taking into account the differences in the physiology of legume seeds and cereal grains, to produce malts with better technological properties, such as lower filtration time, higher wort volume, and higher wort extract content. It was even possible to modify the malting and mashing technology of the green lentil to such an extent that it was possible to produce beers with typical extract and alcohol content, volatile composition, and acceptable taste [8,12]. As it was possible to create a malting and mashing technology for the lentil seeds, the goal of this study is to analyze pea malts created using modified malting technology (more adequate for the legume seed physiology, by increasing moisture content of the germinated seeds), as the pea malts produced in a previous study were also characterized with one of the best properties, such as quick starch saccharification, high wort extract content, high extract volume, and low viscosity [5]. To expand the scope of the study, twelve different *Pisum sativum* varieties were used. Additionally, the study aimed not only to determine the technological properties of the malts, but also to analyze changes in the volatile composition of the acquired malts to assess whether malting can be a viable tool in improving/changing pea odor.

## 2. Materials and Methods

### 2.1. Raw Material

Raw material used in this study were seeds of twelve *Pisum sativum* varieties: ‘Ambrosia’, ‘Biznes’, ‘Cud Kelvedonu’, ‘Gloriosa’, ‘Iłówiecki’, ‘Izolda’, ‘Jantar’, ‘Kantata’, ‘Polar Nochowski’, ‘Primavil’, ‘Sześciotygodniowy’, and ‘Walor’ acquired from Legutko company (Jutrosin, Poland). Abbreviations of the varieties are as follows: Am—‘Ambrosia’, Biz—‘Biznes’, Cud—‘Cud Kelvedonu’, Glo—‘Gloriosa’, Ilo—‘Iłówiecki’, Izo—‘Izolda’, Jan—‘Jantar’, Kan—‘Kantata’, Pol—‘Polar Nochowski’, Pri—‘Primavil’, Sze—‘Sześciotygodniowy’, and Wal—‘Walor’, and they indicate congress worts produced from malts of such variety. Addition of ‘-A’ to the abbreviation means congress worts produced from malt of mentioned variety with addition of alpha-amylase. Addition of ‘-Seed’ to the abbreviation indicates unmalted seeds of such variety. Addition of ‘-Malt’ to the abbreviation indicates malts produced from such variety.

### 2.2. Malting Technology

Prior to malting, the moisture content of the pea seeds was analyzed and measured using the Brabender MT moisture analyzer (Brabender GmbH & Co, Duisburg, Germany) and it was in the range 10.4–12.2% (*w*/*w*). Sixty grams of pea seeds of each variety were loaded into stainless-steel perforated containers, which were previously disinfected in a laboratory dryer (200 °C, 2 h, Memmert UF110 Plus, Memmert GmbH + Co. KG, Schwabach, Germany) and weighed. Three containers of each pea variety were prepared. Containers with pea seeds were weighed and from now will be referenced as a ‘malting kits’. Malting kits were submerged in tap water for 6 h (15 °C), after which they were removed from the water, strained, and weighed again to determine the water content of the pea seeds after steeping. Moisture content of the pea seeds increased to 58–60% (*w*/*w*). After steeping, malting kits were transferred to germination chamber (KK 240 Smart Pro, POL-EKO-APARATURA Sp. J., Wodzisław Śląski, Poland) set at 15 °C and 90% relative humidity. Pea seeds were germinated in a germination chamber for 120 h. Every 24 h of the germination, peas were mixed and sprayed with sterile distilled water. After 120 h of germination, malting kits were transferred to a Memmert UF110 Plus laboratory dryer and dried using the following temperature program: 50 °C (17 h and 50 min), ramp up to 65 °C (10 min), 65 °C (2 h and 50 min), and ramp up to 82 °C (10 min), 82 °C (2 h). After drying, malts of each pea variety, from three different containers, were mixed together and transferred to polypropylene containers, which were then closed tightly to mitigate water adsorption. After malts cooled, they were manually deculmed and ground using Buhler MIAG disc mill with the gap between discs set at 0.2 mm. This malting procedure allowed for the production of 12 malts.

### 2.3. Analysis of Malt Technological Properties—Congress Mashing

The twelve pea malts produced during this study were mashed using the standard Analytica EBC congress mashing procedure [13]. Subsequent mashing involved the addition of 0.03 cm^3^ alpha-amylase at the start of the mashing at temperature of 45 °C to determine whether alpha-amylase addition would improve the properties of the acquired wort. Enzyme added was alpha-amylase from *Bacillus* sp. (Sigma-Aldrich, St. Louis, MO, USA, ≥1 mg protein per cm^3^; ≥500 units per mg of protein; one unit will liberate 1 mg of maltose from starch in 3 min. at pH 6.9 at 20 °C).

Each mashing was performed in duplicate (two for mashing without alpha-amylase addition, and two for mashing with alpha-amylase addition). During and after the mashing, parameters, such as saccharification time, filtration time, wort volume, wort extract content, wort pH, and wort viscosity were analyzed. Saccharification time, filtration time, and wort volume were analyzed using Analytica EBC procedure, with one repetition per each wort acquired [13]. Wort extract content was analyzed usingAnton Paar DMA 35 densimeter (Anton Paar GmbH, Graz, Austria); wort pH was analyzed using Mettler Toledo MP220 pH meter; wort viscosity was measured using Anton Paar ViscoQC 100 rotational viscometer (Anton Paar GmbH, Graz, Austria). Analysis of wort, pH, and viscosity was performed with three repetitions for each wort acquired.

### 2.4. Analysis of Carbohydrates in Worts

Carrez solution I and II were added to the worts to precipitate the proteins. Worts were then centrifuged (10,000 rpm, 10 min) and supernatants were diluted using ultra-pure water, after which they filtered through syringe nylon filters (0.45 µm pore size) to chromatographic vials. The samples were then analyzed using a HPLC Prominence system (Shimadzu Corp., Kyoto, Japan) equipped with a Rezex ROA-Organic Acid H + column (300 × 4.6 mm; Phenomex, Torrance, CA, USA). The following measurement parameters were used: sample volume was equal to 20 mm^3^; oven temperature was equal to 60 °C; mobile phase flow rate: 0.6 mL/min; mobile phase was 0.005 M H_2_SO_4_, and the detection temperature: 50 °C.

### 2.5. Analysis of Volatile Comopounds in Pea Seeds and Pea Malts

Gas chromatography and mass spectrometry (GC-MS) was used to analyze volatile compounds in seeds and malts. Firstly, volatiles had to be extracted from the ground seeds/malts to Solid-Phase Microextraction Fiber (SPME). Malts were ground as stated in Section 2.2, not-malted seeds were firstly coarse-ground using IKA A 10 Basic mill, and then using DLFU Buhler MIAG disc mill, as were the malts. The SPME and GC-MS parameters were treated as in Gasiński & Kawa-Rygielska (2022), with small differences: 1 g of ground seed/malt sample was used, and 5 cm^3^ of saturated NaCl solution (20 °C) and 50 ng of internal standard (2-undecanone, 1 mg per 1 dm^3^ of cyclohexane) were added to the 20 cm^3^ headspace vial after the sample; heatplate was set at 25 °C and 1 cm magnetic stirrer was added to the vial [10]. The stirrer rotation was set at 300 rpm. Five min of temperature equilibration was used, after which the fiber was exposed to the sample headspace and volatiles were adsorbed for 40 min. Three analyses were performed for each malt/seed sample.

### 2.6. Data Analysis

One-way ANOVA was used to compare data acquired in this study. Tukey test was used to determine the homogenous groups (α = 0.05). The software used for the data analysis was Statistica 13.3 software(TIBCO Software Inc., Palo Alto, CA, USA). Principal component analysis was used to analyze factors influencing composition of volatiles in pea seeds and malts.

## 3. Results and Discussion

### 3.1. Technological Properties of Pea Malts

The 12 different malts produced from peas were mashed using the standardized congress mashing regime, and additional congress mashing with the addition of alpha-amylase was performed to determine whether enzyme addition can the improve technological properties of the pea malts. Technological properties of the malts analyzed on the basis of congress mashing analysis are presented in Table 1.

During mashing, the starch in the samples mashed without the addition of alpha-amylase did not hydrolyze fully; therefore, saccharification time could not be determined. In the samples mashed with the addition of alpha-amylase, in three mashes (Glo-A, Jan-A, and Pri-A), the starch was completely hydrolyzed after 50 min of mashing at a temp. of 70 °C. Typically, base malts should saccharify in 20 min during the congress mashing regime; therefore, amylolytic activity and/or starch gelatinization temperature of the pea starch is less adequate for the production of the worts than in typical barley malts [2]. A similar property of the pea malt was determined in the previous study about pea malt, in which the pea malt was malted in a malting regime typical for the production of Pilsner malt (barley) [5]. However, previous research about legume malts, such as green lentil malt, has shown that the modification of the mashing regime can be the only necessary step needed to produce beer from malt of this type [12]. Filtration time of the worts produced from malts mashed without the addition of alpha-amylase were in the range of 16–57 min, with the exception of Pri, which has not filtered completely after 120 min (with 120 min being the maximum filtration time during the congress mashing regime). All worts produced during the mashing with the addition of alpha-amylase were filtered fully in 17–46 min, with the exception of Sze-A, which was filtered in 93 min. Filtration time under 60 min is described as ‘normal’, while filtration time of 61–120 min is described as ‘slow’. Alpha-amylase addition improved filtration time of some worts (Cud, Pol, and Pri), but slowed filtration of other (Biz, Ilo). Alpha-amylase addition can decrease the amount of starch present in the wort, can change the amount of dextrins present in the filtered wort, and/or significantly modify the starch–protein matrix of the malt, releasing more proteins, which can substantially hinder the filtration process [2,14]. The composition of the pea malts, pea worts, and particular processes occurring during the mashing need to be analyzed more thoroughly to pinpoint particular phenomena changing the filtration time of the pea malts and can be an interesting avenue of research in the future. Volume of the worts mashed without alpha-amylase addition was in the range of 175–205 cm^3^, with the exception of Pri, which was the lowest (127.5 cm^3^). Alpha-amylase addition generally improved the process of mashing, as the volume of worts produced from malts mashed with the addition of these enzymes was in the range of 195–240 cm^3^, with the exception of Sze-A (160 cm^3^) and in Am-A, Ilo-A, Pol-A, and Pri-A the difference was statistically significant. These results are typical, as the starch has good water adsorption capability; therefore, hydrolysis of this compound generally should allow for the filtration of more wort [2,15]. However, it is still important to point out that legume seeds, due to the high protein content, are typically more water-absorbent than cereal seeds, and the total amount of wort was significantly lower than in the mashing of typical cereal malts, as the normal amount of wort acquired during congress mashing of barley or wheat malts is in the range of 300–340 cm^3^ [8]. Nevertheless, this flaw of pea malts could be easily mitigated in the industrial process by using filter presses, which are commonly used in large-scale breweries [2]. A large discrepancy can be seen in the wort extract content of the worts produced with, and without, the use of alpha-amylase. The worts acquired without the addition of the external enzyme were characterized with extract content in the range of 2.90–4.13% [*w*/*w*], while those produced using alpha-amylase contained 4.87–6.57% [*w*/*w*]. The addition of alpha-amylase was sufficient to increase extract content of the wort acquired using each of the pea malts. In the previous study, about the legume malts produced using the typical malting technology, pea malt wort was characterized with far lower extract content and the addition of alpha-amylase enzyme did not have as substantial an effect as in this study [5]. Therefore, it seems that the modified malting technology (with higher water content of the germinating seeds) is a good method to acquire malts adequate for the production of legume wort characterized with relatively high extract content, but it still needs to be mentioned that typical barley malt worts contain circa 9.0% [*w*/*w*] extract. Lower extract content of the legume malts is most probably the result of the lower starch content and higher protein content of legumes compared to cereals. However, the lower extract of the wort does not have to be seen as a disadvantage for some of the beer types, as the more and more popular low-alcoholic beers are typically produced from the wort with reduced extract content [16,17]. The pH of the worts was in the range 5.89–6.27 and the worts acquired using the alpha-amylase addition were characterized by similar, or slightly higher, pH than their counterparts acquired without the enzyme addition. The most probable result of higher pH in the worts acquired with the use of alpha-amylase is the higher wort volume, which results in a dilution of chemical compounds which ionize in the solution. As the alpha-amylase only hydrolyses starch, and products of starch hydrolysis have a small impact on the pH, and the higher extract content of the worts does not translate to lower pH [2,18]. Other occurrence is probable because of the changes in the wort viscosity. Worts acquired with the addition of alpha-amylase were characterized with higher or lower viscosity, depending on the sample. Typically, the higher extract content of the wort is the main property influencing increased wort viscosity, due to the properties of the dissolved sugars [19]. However, different composition of particular sugars in the wort might drastically change the viscosity, as the carbohydrates containing more than one unit of simple sugar, due to their size, interact with more amount of water and at equal molecular concentrations, sugars with higher degree of polymerization increase the viscosity [19,20,21]. On the other hand, the alpha-amylase can reduce the amount of dextrins with four or more glucose molecules, decreasing viscosity. Also, the previously mentioned starch–protein matrix might be hydrolyzed, releasing substances which also can impact wort viscosity.

Modification of the pea malting regime allowed for the production of pea malts, with improved properties, but still further improvement of the malting and/or mashing might be necessary for the pea malt to be used in the brewing technology.

### 3.2. Concentration of Carbohydrates in Pea Worts

HPLC analysis allowed for the determination of the concentration of glucose, maltose, maltotriose, and dextrins in the worts produced through the congress mashing of pea malts (with and without the alpha-amylase addition). The results of the HPLC analysis are presented in Table 2.

Results of the analysis of carbohydrates in worts are much clearer than the analysis of technological properties. Worts acquired without the alpha-amylase addition were characterized with a significantly reduced concentration of each of the analyzed sugars—glucose, maltose, maltotriose, and dextrins. Average concentration of glucose in the worts acquired without the alpha-amylase was equal to 1.4979 mg/dm^3^; of maltose was equal to 6.0219 mg/dm^3^; of maltotriose was equal to 0.7345 mg/dm^3^; and of dextrins was equal to 1.0335 mg/dm^3^. The addition of alpha-amylase increased the concentration of each particular sugar in every wort produced from the different pea variety malt samples. In the worts acquired using alpha-amylase, the average concentration of glucose was equal to 3.8962 mg/dm^3^; of maltose was equal to 12.1067 mg/dm^3^; of maltotriose was equal to 5.4603 mg/dm^3^; and of dextrins was equal to 10.0462 mg/dm^3^. The average total concentration of all these sugars was equal to 9.2878 mg/dm^3^ in worts acquired without the enzyme addition, while the worts acquired using alpha-amylase contained 31.5094 mg/dm^3^. Out of these sugars, only 8.2543 mg/dm^3^ in worts produced without alpha-amylase were fermentable, while, in worts with alpha-amylase, there were 21.4632 mg/dm^3^. As the amount of fermentable sugars is far lower than in typical cereal-based worts, this can indicate that acquiring beer with a typical 4.5–5% alcohol content from pea malts can be difficult and will need a modified mashing regime and/or higher amount of malt [2]. However, with the current trend in the brewing industry, with more and more non-alcoholic beers or beer with reduced alcohol content being produced, the apparent flaw of the pea malts could be turned into an advantage, as the worts with such an amount of sugars could be fermented into a low-alcoholic beverage using typical brewing technology, without needing specialized equipment for dealcoholization [2,16,17,22].

### 3.3. Analysis of Volatile Compounds in Pea Seeds and Pea Malts

**Figure 1:**
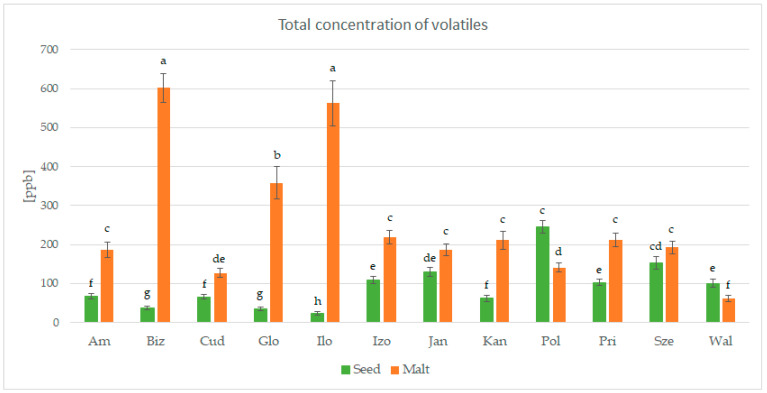
Total concentration of volatiles in pea seeds and malts. Different letters (a, b, …, h) indicate that the means are statistically different.

**Figure 2:**
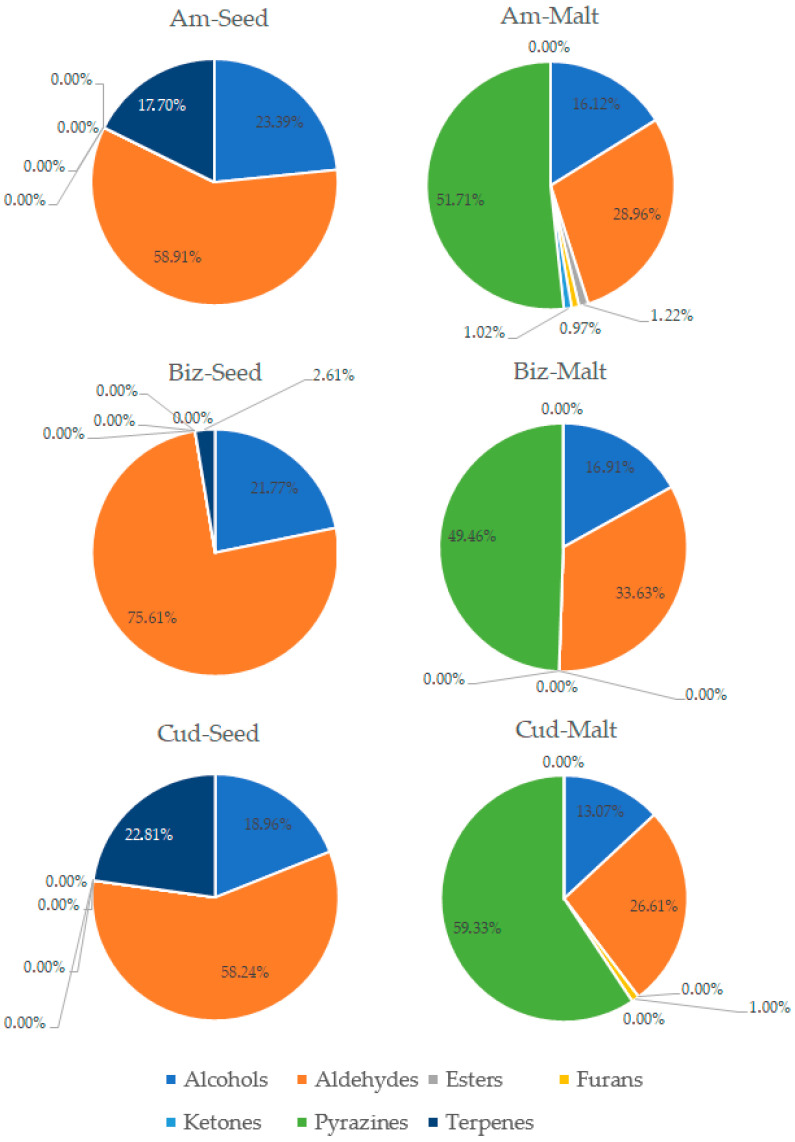
Weight percentage of compounds from different chemical groups in Ambrosia, Biznes, and Cud Kelvedonu variety pea seeds and malts.

**Figure 3:**
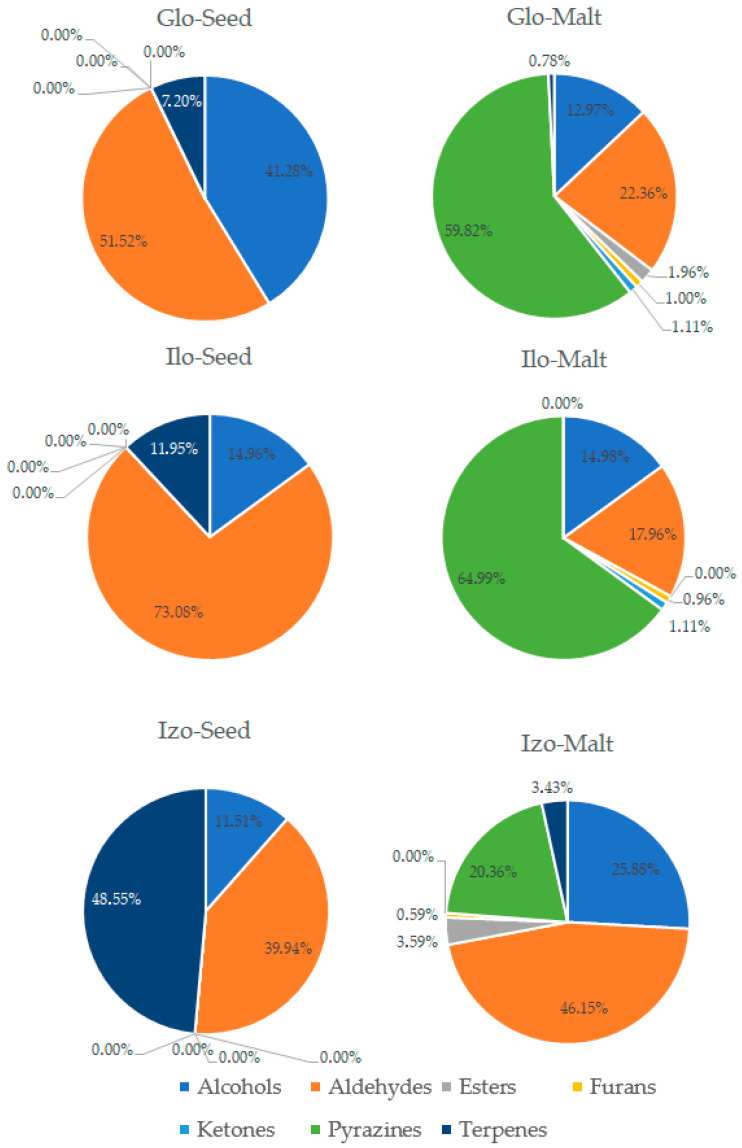
Weight percentage of compounds from different chemical groups in Gloriosa, Iłówiecki, and Izolda variety pea seeds and malts.

**Figure 4:**
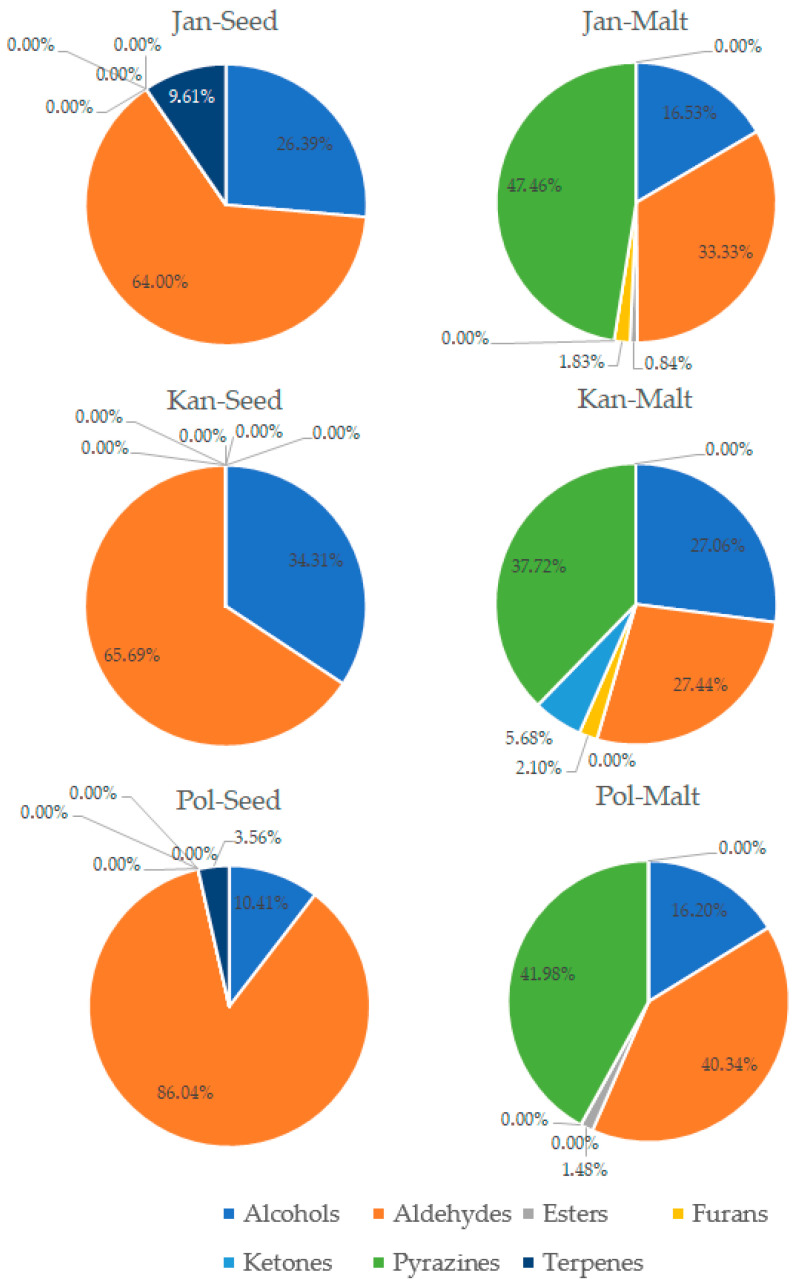
Weight percentage of compounds from different chemical groups in Jantar, Kantata, and Polar Nochowski pea seeds and malts.

**Figure 5:**
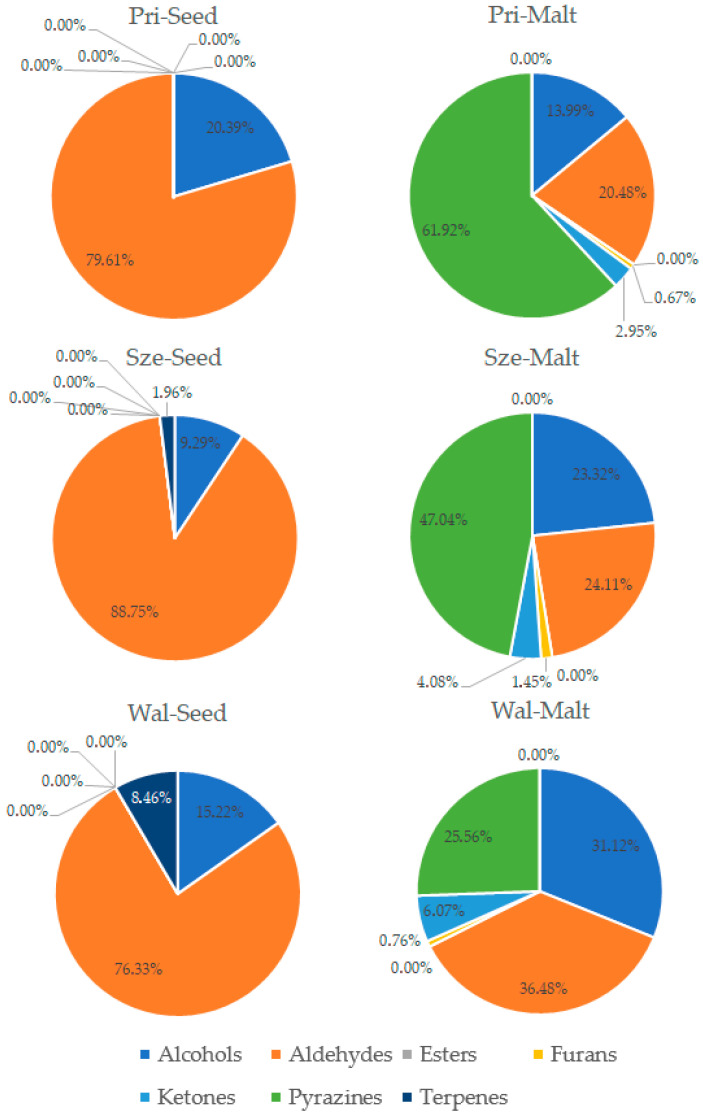
Weight percentage of compounds from different chemical groups in Primavil, Sześciotygodniowy, and Walor variety pea seeds and malts.

**Figure 6:**
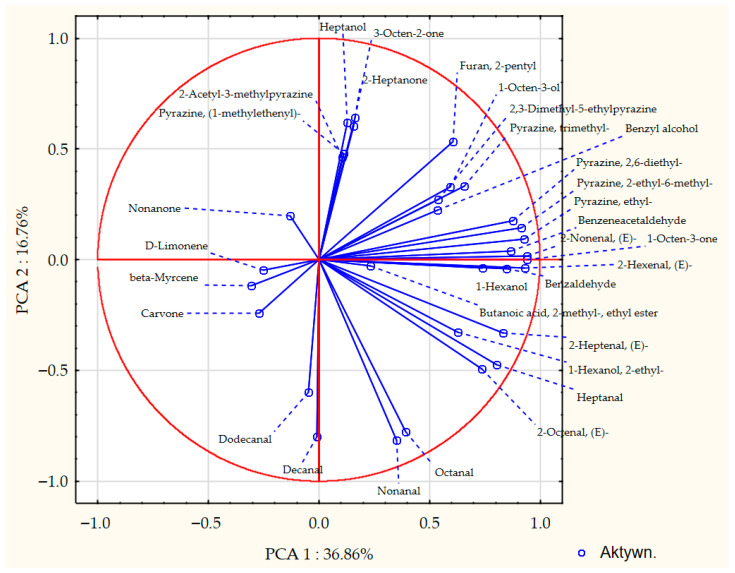
PCA analysis chart for composition of volatile compounds in pea seeds and pea seed malts.

**Figure 7:**
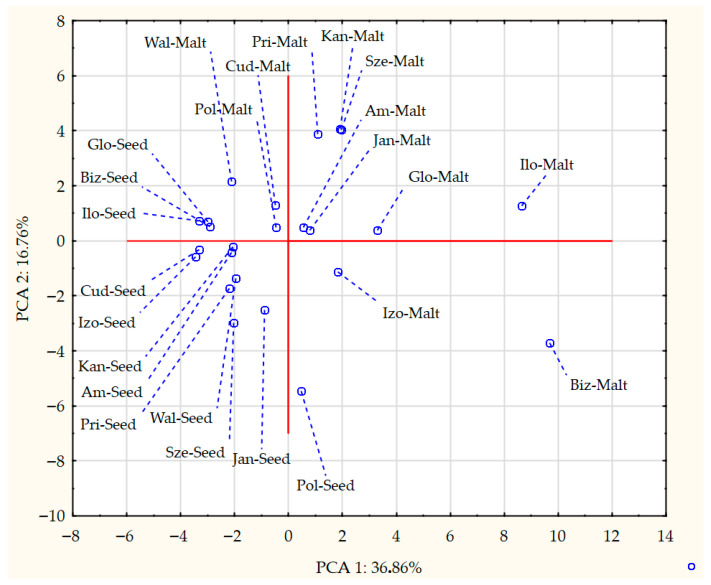
Projection of results of PCA analysis for the concentration of volatile compounds in the pea seed malts and pea seeds.

The process of malting activates plethora of the enzymes present in the seeds, which can modify hundreds of compounds. Each step of the malting process can significantly change the aroma of the product [1,2]. The GC-MS analysis allowed for detection and identification of 32 volatile compounds, 31 in malts and 15 in seeds. Concentration of each particular volatile compound in each malt/seed variety is shown in Supplementary Data, in Tables S1 and S2. Figure 1 shows the total concentration of volatiles in malts and seeds. Figure 2, Figure 3, Figure 4 and Figure 5 show weight percentage of compounds from different chemical groups in the total volatilome of the samples. Figure 6 and Figure 7 show results of the PCA analysis of the concentration of volatile compounds in malts and seeds.

Most of the pea malts were characterized with a greater total concentration of volatiles than unmalted pea seeds, with Pol-Malt and Wal-Malt being the exceptions. The total concentration of volatiles in seeds was in the range from 23.59 to 245.62 ppb. In the malts, the concentration of volatiles ranged from 61.73 to 601.78 ppb. The average increase in the concentration of volatiles due to the malting process was by 652%. However, the greatest increase was noted for the Ilo-Malt, as the concentration of volatile compounds during malting increased almost 24 times. In the malts in which the concentration of volatiles was reduced, the malts contained 57% and 61% of the volatiles present in the seeds. Malts generally contain a far greater amount of volatiles than unmalted seeds/cereals, due to the activity of various enzymes during the process of malting [23]. However, if the generated volatiles have a low boiling point, the temperatures of the kilning process can significantly reduce their content [23,24]. Notwithstanding, the change in total concentration of volatiles is not the main difference between seeds and malts. Substantial changes in the concentration of different chemical groups were noted. Unmalted pea seeds contained just alcohols, ketones, and some terpenes (primarily carvone, D-limonene, and beta-myrcene). In the malts, majority of the volatile compounds were alcohols, aldehydes, and pyrazines. The aldehydes constituted a smaller percentage of the total concentration of volatiles in the malts than in the seeds, but, generally, the total concentration of aldehydes in malts was greater than in seeds. This is typical during the production of malts, as some of the enzyme groups active during the germination process are lipases and lipooxygenases, and this activity generates a large amount of aldehydes from the fat present in the cereals [25]. Previous research about malted lentils have shown similar results: the lentil malts were characterized with far greater concentration of aldehydes than unmalted lentils [10]. However, it is important to note that the concentration of these compounds is not as substantial as to be classified as a malt flaw, as typical cereal malts contain a greater concentration of these components (especially trans-2-nonenal) than the pea malts acquired in this study [26,27,28]. The pea malt volatilome was also characterized by a large contribution of pyrazines. The presence of pyrazines is a typical result of the Maillard reactions due to the presence of heat during the drying process [29,30]. Amylases and proteases release amino acids and monosaccharides during germination, which then can be a substrate for the formation of pyrazines. A small amount of Maillard reaction compounds are necessary for malts, as beer aroma (depending on the beer style) should contain toasty, grainy, and caramelly notes; therefore, a large amount of pyrazines might be beneficial, especially if pea malts were to be used as an addition to light cereal malts [1,2,29]. The PCA analysis has also shown that there are some groups of compounds in which concentration correlates with each other. One such group is heptanol, 3-octen-2-one, 2-heptanone, 2-acetyl-3-methylpyrazine, and 1-methylethenylpyrazine. The second group of compounds in which concentrations correlated was 2-pentylfuran, 1-octen-3-ol, 2,3-dimethyl-5-ethylpyrazine, and pyrazine, trimethyl-, and benzyl alcohol. The third group of such compounds was larger and consisted of pyrazine, 2,6-diethyl-; pyrazine, 2-ethyl-6-methyl-; pyrazine, ethyl-; benzeneacetalehyde; 1-octen-3-one; (E)-2-nonenal, benzaldehyde, and 1-hexanol. The fourth group was nonanal and octanal; the fifth group was decanal and dodecanal, while sixth group consisted of D-limonene, beta-myrcene, and carvone. Concentration of the compounds from the sixth group correlated negatively with concentration of most pyrazines. This is most probably related to the high volatility of terpenes, which can be lost during the drying process or during the prolonged oxidative conditions [10,30]. Similar results were seen already in malting other legumes. In the study about malted lentils, the green lentil was characterized with a high concentration of terpenes, but malts from these seeds contained far less of these components [10]. The most probable reason for the different correlating groups of compounds were the different substrates or enzymes needed for their production. The first group, 2-heptanone, 3-octen-2-one, heptanol, 2-acetyl-3-methylpyrazine, and 1-methylethenylpyrazine, is most probably created by a few distinct pathways: Maillard reaction for pyrazines, lipid oxidation for 2-heptanone, LOX oxidation of linoleic acid for 3-octen-2-one, and reduction of 2-heptanone to heptanol [31,32]. While the correlation between heptanol and 2-heptanone seems obvious, the lack of correlation between these particular two pyrazines and the other pyrazines present in the samples is harder to explain. Most pyrazines are formed due to the Maillard reaction between aminoacids and reducing sugars; therefore, one could expect that the increase in particular pyrazine content should be correlating with the increase in other pyrazines [33]. However, different pyrazines can be generated from different amino acids, which can be cleaved from proteins in different quantities in the pea malts. Also, different pyrazines are generated in greater or smaller quantities, depending on the temperature used [34,35,36]. Volatility of the pyrazines is also significantly different; therefore, during prolonged heating, the concentration of some pyrazines could greatly diminished, while others could remain present in the sample [37]. The second group of compounds, 2-pentylfuran, 1-octen-3-ol, 2,3-dimethyl-5-ethylpyrazine, and pyrazine, trimethyl-, and benzyl alcohol also share similarities for their origin. As already mentioned, pyrazines are formed through Maillard reaction, but 1-octen-3-ol and 2-pentylfuran are formed from linoleic acid through lipid oxidation. Benzyl alcohol is typically formed from benzeneacetaldehyde (phenylacetaldehyde), which is formed through the degradation of phenylalanine [38,39,40,41]. However, as the substrates for these compounds are not the same, their correlation might be incidental or related to the temperature of the formation, the volatility of these compounds, or similar availability of the substrates due to the enzyme activity and concentration of different amino acids and lipids in the pea seeds. The third group also shares similar formation mechanisms as the second group. The fourth group of compounds, nonanal and decanal, have a slightly different origin, as they are formed from an oleic acid rather than linoleic acid [42]. Pea seeds can contain vastly different concentrations of oleic and linoleic acid (depending on the cultivar), which can possibly explain the change in the concentration of decanal and nonanal compared to the other compounds [43]. The concentration of dodecanal correlated more with the concentration of decanal, than nonanal and octanal. However, dodecanal and decanal can be formed from palmitoleic acid and lauric acid, which are different substrates than for nonanal and octanal [44,45]. All the gathered data indicates that the malting procedure is a viable strategy to modify the composition of volatiles in peas and, therefore, could be used as a part of a process which would result in novel, pea-based food products with a modified aroma.

## 4. Conclusions

Some varieties of peas are more adequate for the production of malts, but, generally, malted peas are characterized by worse technological parameters than typical barley malt, albeit the addition of alpha-amylase to the mashing significantly improved the parameters of the worts, such as wort volume and wort extract. However, it is possible to acquire a gluten-free wort with an adequate concentration of fermentable sugars, which could be used for the production of beer with reduced alcohol content. Furthermore, the malting procedure significantly changes the composition of volatiles in pea malts, increasing the amount of ketones and pyrazines present in the volatilome of the malt, while reducing, at the same time, the amount of terpenes. Pea malts can, therefore, possibly be an interesting substrate for a whole array of novel, legume-based products characterized with a modified odor.

## Figures and Tables

**Table 1:** Technological properties of pea malts.

Sample ^1^	Saccharification Time [min]	Filtration Time [min]	Wort Volume [cm^3^]	Wort Extract [%*w*/*w*]	Wort pH	Wort Viscosity [mPa·s]
Am	X	16 ± 1 i	205 ± 5 c	3.60 ± 0.08 j	6.05 ± 0.02 g	1.679 ± 0.030 c
Biz	X	21 ± 1 h	200 cd	4.13 ± 0.05 h	6.06 ± 0.01 g	1.555 ± 0.064 de
Cud	X	48 ± 2 d	195 ± 5 d	3.93 ± 0.05 i	6.09 ± 0.03 ef	1.660 ± 0.072 c
Glo	X	16 ± 1 i	205 c	3.97 ± 0.05 i	6.20 ± 0.03 c	1.466 ± 0.042 gh
Ilo	X	23 ± 2 h	195 ± 5 d	3.17 ± 0.05 l	6.26 ± 0.02 ab	1.525 ± 0.019 e
Izo	X	30 ± 3 g	195 ± 5 d	3.27 ± 0.05 k	5.89 ± 0.03 hi	1.867 ± 0.027 a
Jan	X	18 ± 1 i	207.5 ± 2.5 bc	3.90 ± 0.08 i	6.28 ± 0.01 a	1.503 ± 0.004 f
Kan	X	23 ± 1 h	207.5 ± 2.5 bc	3.67 ± 0.05 j	6.19 ± 0.01 c	1.383 ± 0.056 i
Pol	X	31 ± 2 g	205 ± 5 c	4.10 ± 0.08 h	6.13 ± 0.02 e	1.484 ± 0.008 g
Pri	X	120 a	127.5 ± 2.5 g	3.90 ± 0.08 i	6.29 ± 0.02 a	1.657 ± 0.031 c
Sze	X	57 ± 1 c	175 ± 5 e	3.17 ± 0.05 l	6.23 ± 0.02 bc	1.686 ± 0.023 c
Wal	X	29 ± 1 g	205 ± 5 c	2.90 ± 0.08 m	5.98 ± 0.03 h	1.560 ± 0.053 e
Am-A	X	17 ± 1 i	215 ± 5 b	5.57 ± 0.05 cd	6.09 ± 0.02 f	1.485 ± 0.008 g
Biz-A	X	28 ± 2 g	202.5 ± 2.5 cd	5.70 ± 0.08 c	6.11 ± 0.02 e	1.484 ± 0.009 g
Cud-A	X	38 ± 1 e	200 ± 5 cd	5.33 ± 0.05 e	6.03 ± 0.02 g	1.600 ± 0.049 d
Glo-A	50a	17 ± 1 i	210 ± 5 bc	5.47 ± 0.05 d	6.20 ± 0.02 c	1.533 ± 0.041 ef
Ilo-A	X	35 ± 1 f	240 ± 10 a	6.27 ± 0.05 b	6.27 ± 0.02 ab	1.590 ± 0.023 d
Izo-A	X	46 ± 2 d	195 ± 5 d	5.00 ± 0.03 f	5.89 ± 0.01 i	1.750 ± 0.079 bc
Jan-A	50a	16 ± 1 i	205 ± 5 c	5.37 ± 0.05 de	6.26 ± 0.02 ab	1.465 ± 0.004 h
Kan-A	X	21 ± 1 h	205 ± 5 c	5.60 ± 0.03 cd	6.16 ± 0.02 d	1.469 ± 0.008 h
Pol-A	X	29 ± 1 g	210 b	5.57 ± 0.07 cd	6.11 ± 0.02 e	1.608 ± 0.041 d
Pri-A	50a	28 ± 1 g	230 ± 10 a	6.57 ± 0.07 a	6.25 ± 0.01 b	1.507 ± 0.020 f
Sze-A	X	93 ± 3 b	160 ± 10 e	6.27 ± 0.05 b	5.97 ± 0.02 h	1.812 ± 0.005 b
Wal-A	X	30 ± 2 g	200 cd	4.87 ± 0.05 g	5.93 ± 0.03 hi	1.810 ± 0.034 b

^1^ Values are expressed as mean ± standard deviation. Mean values with different letters (a, b, …, j) within the same column are statistically different (*p*-value < 0.05). Green color indicates highest mean values in the column, while red indicates lowest mean value in the column. X—stands for no saccharification of analyzed sample. Abbreviations of the samples are described in Section 2.1 of the manuscript.

**Table 2:** Concentration of carbohydrates in pea worts.

Sample	Glucose Concentration [mg/dm^3^]	Maltose Concentration [mg/dm^3^]	Maltotriose Concentration [mg/dm^3^]	Dextrin Concentration [mg/dm^3^]
Am	2.0224 ± 0.1096 f	8.5777 ± 0.5284 ef	0.3898 ± 0.0461 i	1.1021 ± 0.1100 g
Biz	1.5674 ± 0.1178 fg	6.4593 ± 0.5116 g	0.9856 ± 0.1248 g	0.9646 ± 0.1062 gh
Cud	1.4249 ± 0.1103 g	5.400 ± 0.5116 h	0.3642 ± 0.0311 i	0.9011 ± 0.1118 h
Glo	1.9063 ± 0.1273 f	8.1664 ± 0.5093 f	1.3423 ± 0.0252 f	1.3834 ± 0.2074 f
Ilo	1.2816 ± 0.1198 h	5.4738 ± 0.6421 h	0.7322 ± 0.1002 h	0.8933 ± 0.0944 h
Izo	1.0458 ± 0.1028 i	4.3094 ± 0.6356 i	0.3252 ± 0.0344 i	0.8660 ± 0.0885 h
Jan	1.7162 ± 0.1011 f	7.7152 ± 0.5106 f	0.9146 ± 0.1116 g	1.2423 ± 0.2112 f
Kan	1.4632 ± 0.1054 g	5.8443 ± 0.6146 gh	1.3518 ± 0.0206 f	1.2034 ± 0.1857 fg
Pol	1.7749 ± 0.1279 f	7.2586 ± 0.4328 fg	0.7253 ± 0.1124 h	1.0349 ± 0.1621 gh
Pri	1.3335 ± 0.1054 h	5.5951 ± 0.7023 h	0.6986 ± 0.1214 h	0.9375 ± 0.1018 gh
Sze	1.3126 ± 0.1099 h	5.2802 ± 0.6062 hi	0.6683 ± 0.0621 h	0.8335 ± 0.0983 h
Wal	1.1256 ± 0.1136 i	4.1868 ± 0.6172 i	0.3161 ± 0.0383 i	1.0394 ± 0.1416 gh
Am-A	4.1447 ± 0.1121 b	13.1858 ± 0.3311 b	5.2627 ± 0.3137 c	10.8356 ± 0.3441 c
Biz-A	4.5375 ± 0.1147 a	14.8139 ± 0.4084 a	4.8229 ± 0.5025 cd	9.7117 ± 0.4052 d
Cud-A	3.7745 ± 0.1033 d	11.2798 ± 0.4206 cd	4.1326 ± 0.3223 e	10.9304 ± 0.4140 c
Glo-A	4.3621 ± 0.1645 ab	14.6471 ± 0.3363 a	4.7837 ± 0.3279 d	9.7221 ± 0.4570 d
Ilo-A	3.8318 ± 0.1045 cd	10.6672 ± 0.3123 d	6.8807 ± 0.4169 b	6.3376 ± 0.4453 e
Izo-A	3.1349 ± 0.1103 e	9.0223 ± 0.6127 e	4.7699 ± 0.4127 d	13.8059 ± 0.3281 a
Jan-A	4.0424 ± 0.1282 bc	14.1430 ± 0.5642 ab	4.5585 ± 0.3827 de	9.0408 ± 0.5386 d
Kan-A	4.0086 ± 0.1099 bc	13.5496 ± 0.4710 b	4.9639 ± 0.4081 cd	10.7546 ± 0.3158 c
Pol-A	4.1205 ± 0.1211 bc	11.9284 ± 0.4096 c	7.8676 ± 0.4297 a	6.8969 ± 0.3033 e
Pri-A	3.9144 ± 0.1190 c	13.1605 ± 0.3208 b	5.3336 ± 0.4119 c	12.0555 ± 0.4771 b
Sze-A	3.6933 ± 0.1152 d	9.8168 ± 0.6212 de	7.1031 ± 0.5347 ab	6.6166 ± 0.4030 e
Wal-A	3.1894 ± 0.1139 e	9.0664 ± 0.5334 e	5.0445 ± 0.4330 c	13.8469 ± 0.2080 a

Values are expressed as mean ± standard deviation. Mean values with different letters (a, b, …, i) within the same column are statistically different (*p*-value < 0.05). Green color indicates highest mean values in the column, while red indicates lowest mean value in the column. Abbreviations of the samples are described in Section 2.1 of the manuscript.

## Data Availability

The original contributions presented in this study are included in the article/Supplementary Material. Further inquiries can be directed to the corresponding author.

## Supplementary Materials

The following supporting information can be downloaded at the following address: https://www.mdpi.com/article/10.3390/foods14183224/s1, Table S1: Concentration of volatiles in pea malts; Table S2: Concentration of volatiles in pea seeds, Table S3: Mass percentage of different chemical groups in total volatile compound concentration in pea malts and seeds.

## References

[1] Bamforth C., Bamforth C. (2009). Grain to Glass: The Basics of Malting and Brewing. Beer: Tap into the Art and Science of Brewing.

[2] Kunze W. (2019). Technology Brewing and Malting.

[3] Salamon A., Kowalska H., Ignaczak A., Marzec A., Kowalska J., Szafrańska A. (2023). Characteristics of Oat and Buckwheat Malt Grains for Use in the Production of Fermented Foods. Foods.

[4] Chawanda E.T., Manhokwe S., Jombo T.Z., Mugadza D.T., Njini M., Manjeru P. (2022). Optimisation of Malting Parameters for Quinoa and Barley: Application of Response Surface Methodology. J. Food Qual..

[5] Gasiński A., Błażewicz J., Kawa-Rygielska J., Śniegowska J., Zarzecki M. (2021). Analysis of Physicochemical Parameters of Congress Worts Prepared from Special Legume Seed Malts, Acquired with and without Use of Enzyme Preparations. Foods.

[6] Trummer J., Watson H., De Clippeleer J., Poreda A. (2021). Brewing with 10% and 20% Malted Lentils—Trials on Laboratory and Pilot Scales. Appl. Sci..

[7] Meena R.S., Lal R., Meena R.S., Das A., Yadav G.S., Lal R. (2018). Legumes and Sustainable Use of Soils. Legumes for Soil Health and Sustainable Management.

[8] Cheng S., Langrish T.A.G. (2025). A Review of the Treatments to Reduce Anti-Nutritional Factors and Fluidized Bed Drying of Pulses. Foods.

[9] Gasiński A., Kawa-Rygielska J., Mikulski D., Kłosowski G. (2022). Changes in the raffinose family oligosaccharides content in the lentil and common bean seeds during malting and mashing processes. Sci. Rep..

[10] Gasiński A., Kawa-Rygielska J. (2023). Malting—A method for modifying volatile composition of black, brown and green lentil seeds. PLoS ONE.

[11] Gasiński A., Noguera-Artiaga L., Kawa-Rygielska J. (2025). Influence of Malted Chickpea on the Composition of Volatiles in Hummus. Molecules.

[12] Gasiński A., Kawa-Rygielska J. (2024). Assessment of green lentil malt as a substrate for gluten-free beer brewing. Sci. Rep..

[13] EBC—Analytica (1998). 4.5.1 Extract of Malt: Congress Mash.

[14] Cai G., Li X., Zhang C., Zhang M., Lu J. (2016). Dextrin as the main turbidity components in wort produced from major malting barley cultivars of Jiangsu province in China. J. Inst. Brew..

[15] Barreiro J.A., Fernández S., Sandoval A.J. (2003). Water sorption characteristics of six row barley malt (*Hordeum vulgare*). LWT-Food Sci. Technol..

[16] Piornos J.A., Koussissi E., Balagiannis D.P., Brouwer E., Parker J.K. (2023). Alcohol-free and low-alcohol beers: Aroma chemistry and sensory characteristics. Compr. Rev. Food Sci. Food Saf..

[17] Ivanov K., Petelkov I., Shopska V., Denkova R., Gochev V., Kostov G. (2016). Investigation of mashing regimes for low-alcohol beer production. J. Inst. Brew..

[18] Li G., Liu F., Kun-Farkas G., Kiss Z. (2015). Technological factors influencing buffering capacity of wort. J. Am. Soc. Brew. Chem..

[19] Sorokin S.A., Novoselov A.G., Kuznetsov A.Y., Baranov I.V., Rumiantceva O.N., Mironova D.Y., Kiliashov A.A., Kravtsova E.V. (2024). Comprehensive studies of the physical and thermophysical properties of wort. BIO Web Conf..

[20] Juszczak L., Gałkowska D., Witczak T., Fortuna T. (2013). Effect of Maltodextrins on the Rheological Properties of Potato Starch Pastes and Gels. Int. J. Food Sci..

[21] Sadosky P., Schwarz P.B., Horsley R.D. (2002). Effect of arabinoxylans, beta-glucans, and dextrins on the viscosity and membrane filterability of a beer model solution. J. Am. Soc. Brew. Chem..

[22] Bellut K., Arendt E.K. (2019). Chance and challenge: Non-*saccharomyces* yeasts in nonalcoholic and low alcohol beer brewing–A review. J. Am. Soc. Brew. Chem..

[23] Prado R., Gastl M., Becker T. (2021). Aroma and color development during the production of specialty malts: A review. Compr. Rev. Food Sci. Food Saf..

[24] Hodges M.D., Fitzgerald N. (2020). Investigating the Potential of an In-Situ Method for Monitoring the Malting of Barley Using Solid Phase Microextraction with a Portable Gas Chromatography Mass Spectrometry Instrument. Beverages.

[25] Guido L.F., Ferreira I.M. (2023). The Role of Malt on Beer Flavour Stability. Fermentation.

[26] Gasiński A., Pytlarz E., Hamkało O., Kawa-Rygielska J. (2023). Technological properties and composition of volatile compounds in winter wheat malts grown with addition of seed meals into soil. Sci. Rep..

[27] Filipowska W., Jaskula-Goiris B., Ditrych M., Bustillo Trueba P., De Rouck G., Aerts G., Powell C., Cook D., De Cooman L. (2021). On the contribution of malt quality and the malting process to the formation of beer staling aldehydes: A review. J. Inst. Brew..

[28] Svoboda Z., Mikulíková R., Běláková S., Benešová K., Marová I., Nesvadba Z. (2011). Optimization of Modern Analytical SPME and SPDE Methods for Determination of Trans-2-nonenal in Barley, Malt and Beer. Chromatographia.

[29] Hellwig M., Henle T. (2020). Maillard Reaction Products in Different Types of Brewing Malt. J. Agric. Food Chem..

[30] Kern S., Granier T., Dkhil H., Haupt T., Ellis G., Natsch A. (2014). Stability of limonene and monitoring of a hydroperoxide in fragranced products. Flavour Fragr. J..

[31] Grebenteuch S., Kanzler C., Klaußnitzer S., Kroh L.W., Rohn S. (2021). The Formation of Methyl Ketones during Lipid Oxidation at Elevated Temperatures. Molecules.

[32] Chen J., Zhang L., Guo X., Qiang J., Cao Y., Zhang S., Yu X. (2025). Influence of triacylglycerol structure on the formation of lipid oxidation products in different vegetable oils during frying process. Food Chem..

[33] Yu H., Zhang R., Yang F., Xie Y., Guo Y., Yao W., Zhou W. (2021). Control strategies of pyrazines generation from Maillard reaction. Trends Food Sci. Technol..

[34] Liu X., Quan W. (2024). Progress on the Synthesis Pathways and Pharmacological Effects of Naturally Occurring Pyrazines. Molecules.

[35] Xia X., Zhou T., Zhang H., Cui H., Zhang F., Hayat K., Zhang X., Ho C.-T. (2023). Simultaneously Enhanced Formation of Pyrazines and Furans during Thermal Degradation of the Glycyl-l-Glutamine Amadori Compound by Selected Exogenous Amino Acids and Appropriate Elevated Temperatures. J. Agric. Food Chem..

[36] Wang F., Shen H., Yang X., Liu T., Yang Y., Zhou X., Zhao P., Guo Y. (2021). Effect of free amino acids and peptide hydrolysates from sunflower seed protein on the formation of pyrazines under different heating conditions. RSC Adv..

[37] Ma Y.J., Wu J.H., Li X., Xu X.B., Wang Z.Y., Wu C., Du M., Song L. (2019). Effect of alkyl distribution in pyrazine on pyrazine flavor release in bovine serum albumin solution. RSC Adv..

[38] Hidalgo F.J., Zamora R. (2016). Amino Acid Degradations Produced by Lipid Oxidation Products. Crit. Rev. Food Sci. Nutr..

[39] Wietstock P.C., Kunz T., Methner F.J. (2016). Relevance of Oxygen for the Formation of Strecker Aldehydes during Beer Production and Storage. J. Agric. Food Chem..

[40] Adams A., Bouckaert C., Van Lancker F., De Meulenaer B., De Kimpe N. (2011). Amino acid catalysis of 2-alkylfuran formation from lipid oxidation-derived α,β-unsaturated aldehydes. J. Agric. Food Chem..

[41] Matsui K., Sasahara S., Akakabe Y., Kajiwara T. (2003). Linoleic acid 10-hydroperoxide as an intermediate during formation of 1-octen-3-ol from linoleic acid in Lentinus decadetes. Biosci. Biotechnol. Biochem..

[42] Cao J., Jiang X., Chen Q., Zhang H., Sun H., Zhang W.-M., Li C. (2019). Oxidative stabilities of olive and camellia oils: Possible mechanism of aldehydes formation in oleic acid triglyceride at high temperature. LWT Food Sci. Technol..

[43] Villalobos Solis M.I., Patel A., Orsat V., Singh J., Lefsrud M. (2013). Fatty acid profiling of the seed oils of some varieties of field peas (Pisum sativum) by RP-LC/ESI-MS/MS: Towards the development of an oilseed pea. Food Chem..

[44] Gao P., Bao Y., Wang S., Lei L., Wang B., Xiao L., Cheng K., Wang Y., Zhang S., Dong L. (2023). Mechanism of palmitoleic acid oxidation into volatile compounds during heating. Flavour Fragr. J..

[45] Behrends T., Schmid J., Blank L.M., Buhler B. (2012). Biotransformation of Fatty Acids to Fatty Aldehydes.

